# Estimating the burden of healthcare-associated infections caused by selected multidrug-resistant bacteria Finland, 2010

**DOI:** 10.1186/2047-2994-1-33

**Published:** 2012-10-19

**Authors:** Mari Kanerva, Jukka Ollgren, Antti J Hakanen, Outi Lyytikäinen

**Affiliations:** 1 Epidemiologic Surveillance and Control Unit, Department of Infectious Disease Surveillance and Control, National Institute for Health and Welfare (THL), P.O. Box 30, FI-00271, Helsinki, Finland; 2 Helsinki University Central Hospital, Department of Medicine, Division of Infectious Diseases, POB 348, FIN-00029, HUS, FINLAND; 3 Unit of Antimicrobial Resistance, Department of Infectious Disease Surveillance and Contro, National Institute for Health and Welfare (THL), P.O. Box 57, FI-20521, Turku, Finland

**Keywords:** Multidrug-resistant microbes, Healthcare-associated infections, Burden of HAI, Infection control, Resource allocation

## Abstract

**Background:**

Knowledge of the burden of healthcare-associated infections (HAI) and antibiotic resistance is important for resource allocation in infection control. Although national surveillance networks do not routinely cover all HAIs due to multidrug-resistant bacteria, estimates are nevertheless possible: in the EU, 25,000 patients die from such infections annually. We assessed the burden of HAIs due to multidrug-resistant bacteria in Finland in 2010.

**Methods:**

By combining data from the National Infectious Disease Registry on the numbers of bacteremias caused by *Staphylococcus aureus*, *Enterococcus faecium*, *Escherichia coli*, *Klebsiella pneumoniae, Enterobacter* spp., *Pseudomonas aeruginosa* and *Acinetobacter* spp.*,* and susceptibility data from the National Antimicrobial Resistance Network and the Finnish Hospital Infection Program, we assessed the numbers of healthcare-associated bacteremias due to selected multidrug-resistant bacteria. We estimated the number of pneumonias, surgical site and urinary tract infections by applying the ratio of these infections in the first national prevalence survey for HAI in 2005. Attributable HAI mortality (3.2%) was also derived from the prevalence survey.

**Results:**

The estimated annual number of the most common HAIs due to the selected multidrug-resistant bacteria was 2804 (530 HAIs per million), 6% of all HAIs in Finnish acute care hospitals. The number of attributable deaths was 89 (18 per million).

**Conclusions:**

Resources for infection control should be allocated not only in screening and isolation of carriers of multidrug-resistant bacteria, even when they are causing a small proportion of all HAIs, but also in preventing all clinical infections.

## Introduction

According to the analysis of European Centre for Disease Prevention and Control (ECDC) and European Medicines Agency (EMEA) in 2007, an estimated number of deaths attributable to infections due to selected multidrug-resistant bacteria, *Staphylococcus aureus, Enterococcus* spp, *Escherichia coli*, *Klebsiella* spp., *Enterobacter* spp*.* or *Pseudomonas aeruginosa,* in the EU, Iceland and Norway was about 25,000 [1]. Approximately 37,000 patients die as a direct consequence of a hospital-acquired infection and an additional 111,000 die as an indirect consequence of the hospital-acquired infection annually [2]. As deaths due to resistant microbes are generally associated with healthcare-associated infections (HAI), antibacterial resistance could be responsible for up to half of HAI deaths. ECDC/EMEA analysis also reported the extra healthcare cost and productivity losses due to resistant bacteria in the EU and it was at least 1.5 billion each year [1].

These estimates give an overview of the magnitude of the problem of antibacterial resistance and HAI in Europe and are important for policymakers and administrators who make resource allocation for infection control. However, the proportion of multidrug-resistant isolates among these bacteria varies between countries, and the data cannot be applied directly to different EU countries.

Earlier we estimated that annually approximately 6% of all patients in acute care hospitals in Finland get at least one HAI, and 3.2% of HAI patients die as a direct or indirect consequence of infection [3]. In the national prevalence survey in 2005, only 1.5% of microbiologically confirmed HAIs were due to tobramycin-resistant *Pseudomonas aeruginosa*, methicillin resistant *S. aureus* (MRSA) or extended spectrum β-lactamase (ESBL) producing *Enterobacteriacae* bacteria [4]. Thus most HAI deaths in Finland are most probably due to non-multidrug-resistant bacteria.

In Finland, several tools are available for the surveillance of antibacterial resistance, some of which are nationwide and others sentinel. However, these data sources cannot directly serve to assess the burden of HAI due to multidrug-resistant bacteria, since laboratory-based surveillance does not distinguish between colonisation and clinical infections, and since among clinical infections, only bacteremias are surveyed nationwide. By using various national surveillance and survey data, we estimated the number of HAIs and HAI mortality based on a set of the best surveyed multidrug-resistant bacteria in Finland in 2010.

## Methods

Data sources included the following national and sentinel surveillance programmes. The National Infectious Disease Registry (NIDR) collects data on invasive bacterial isolates from blood and cerebrospinal fluid, all isolates of MRSA*,* vancomycin-resistant enterococci (VRE) and all isolates of *E. coli* and *Klebsiella pneumoniae* which are intermediately susceptible (I) or resistant (R) to third generation cephalosporins (potential ESBL producers). Since 2011, *Enterobacteriacae* isolates non-susceptible (I/R) to carbapenems were also surveilled; thus data for 2010 was unavailable. During 2011, however, notifications mainly entailed colonisations, rather than infections. The Finnish study group for antimicrobial Resistance (FiRe) is a network of 24 clinical microbiology laboratories (covering >95% of all clinical laboratories that process blood cultures in Finland) that collect susceptibility data on 15 bacteria. Of the 24 FiRe laboratories, 20 report susceptibility data on the invasive isolates of seven indicator pathogens to the European Antimicrobial Resistance Surveillance System (EARS-Net). The Finnish Hospital Infection Program (SIRO), a sentinel network of 15 hospitals, has surveilled HAI, including nosocomial bloodstream infections (BSI) and surgical site infections (SSI) in selected surgical procedures since 1999. SIRO performed the first national prevalence survey of HAIs in 2005, which covered all HAI types and the outcomes of the study patients in 30 acute care hospitals [4].

The NIDR provided the total number of bacteremia cases caused by *S. aureus*, *Enterococcus faecium*, *E. coli*, *K. pneumoniae, Enterobacter* spp., *P. aeruginosa* and *Acinetobacter* spp. in 2010 [5]. Resistance percentages to antibiotics were identified as follows: from the NIDR, for MRSA and VRE as well as I/R for third generation cephalosporins in *E. coli* and *K. pneumonia*; from SIRO and FiRe, I/R for amikacin in *P. aeruginosa,* for third generation cephalosporins in *Enterobacter* spp. and for carbapenems in *Acinetobacter spp*. All these resistant bacteria were considered as multidrug-resistant isolates.

The national prevalence survey of HAIs in 2005 provided the number of pneumonias, SSI and urinary tract infections (UTI) in relation to BSI [4].

By applying the above mentioned resistance percentages to the numbers of bacteremias in the NIDR, we estimated the number of BSI cases due to these resistant bacteria. We estimated the number of other common types of HAIs (pneumonia, as well as SSI and UTI) due to these resistant bacteria by multiplying the number of bacteremias in the NIDR by their corresponding ratio in the national prevalence survey.

The dates and causes of death and 28-day attributable mortality due to HAI in the prevalence survey cohort were obtained from the National Population Information System by using the patient’s national identity code, as described earlier [3]. We estimated the number of deaths from these infections by applying an average attributable mortality of 3.2% [3].

We calculated 95% Bayesian confidence intervals (credible intervals) using Markov Chain Monte Carlo simulations. We used coefficients 0.5 and 1.5 to describe uncertainty in the number of BSI due to resistant bacteria as well as in the proportion of other types of infection.

## Results

Percentages of resistance to the antibiotics in blood isolates were: MRSA 1.9% (26/1370), VRE 1.1% (3/278), third generation cephalosporins for *E. coli* 3.4% (112/3211), and for *K. pneumoniae* 3.2% (16/504), and for *Enterobacter* spp. 34.0% of 259 isolates, amikacin for *P. aeruginosa* 5.0% of 318 isolates and carbapenems for *Acinetobacter* spp. 3.3% of 34 isolates (Table 1).

**Table 1:** Observed* of estimated annual numbers of healthcare-associated infections due to multidrug-resistant bacteria

	**Numbers of HAIs (95% confidence interval)**						
**Type of HAI**	**MRSA**	**VRE**	**Third gen ceph I/R**			**Amicacin I/R *P. aeruginosa***	**Carbapenem I/R *Acinetobacter spp.***
			***E. coli***	***K. pneumoniae***	***Enterobacter spp.***		
Blood stream infection	26* (17–37)	3* (1-7)	112* (92–134)	16* (10–25)	88 (47–136)	16 (9–25)	1 (12)
Pneumonia	65 (29–114)	8 (1–20)	280 (142–440)	40 (16–75)	220 (87–415)	40 (16–75)	3 (1-5)
Surgical site infection	127 (58–22)	15 (3–40)	549 (278–862)	78 (33–147)	431 (172–814)	78 (32–147)	5 (2-10)
Urinary tract infection	60 (27–105)	7 (1–16)	258 (131–405)	37 (15–69)	202 (81–381)	37 (15–69)	2 (1-5)
All	278 (159–434)	33 (6–81)	1199 (795–1654)	171 (87–287)	941 (458–1584)	171 (86–287)	11 (5–19)

Footnote: HAI=healthcare-associated infection, MRSA=methicillin resistant Staphylococcus aureus, VRE=vancomycin resistant Enterococcus faecium, I/R = intermediately suscaptible or resistant.

In the prevalence survey among all 753 infections, the numbers of the most common infection types were as follows: 44 (6%) primary BSIs, 110 (15%) pneumonias, 215 (29%) SSIs, 103 (15%) symptomatic UTIs and 281 (37%) other. In relation to BSIs, the corresponding ratio of the other infection types were 110/44=2.5 for pneumonias, 4.9 (215/44) for SSIs and 2.3 (103/44) for UTIs. The estimated annual numbers of these four common types of HAIs due to the resistant bacteria were calculated by multiplying the number of the BSIs by these infection ratios. Thus for example, the estimated number of pneumonias due to MRSA was 26 x 2.5 = 65, and numbers of SSIs and UTIs due to MRSA were 26 x 4.9 = 127 and 26 x 2.3 = 60, respectively (Table 1).

The estimated total annual numbers of the four HAI types due to MRSA, VRE, third generation cephalosporin I/R *E. coli* and *K. pneumoniae* and *Enterobacter* spp*.*, as well as amicacin I/R *P. aeruginosa* and carbapenem I/R *Acinetobacter* spp. were 278, 33, 1199, 171, 941, 171 and 11, respectively (Table 1), for a total of 2804 (530 HAIs caused by resistant bacteria per million population). The estimated total annual number of attributable deaths due to these infections was 89 (18 per million population).

## Conclusions

This study represents the first attempt to estimate the burden of HAIs due to multidrug-resistant bacteria in Finland. The annual number of HAIs in acute care hospitals in Finland is at least 50 000 [3]. Here we show, that at approximately 2800 (6%) of them may be due to multidrug-resistant bacteria. The HAI morbidity and mortality due to multidrug-resistant bacteria is, in Finland, thus far relatively low and lower than the figures in the EU as a whole [1], but shows differences depending on the resistant bacterium. The methodology we used was based on the work presented by the ECDC/EMEA and might be applied also by other countries in their burden assessments [1].

The results we present here are a rough estimate. True figures of the burden of all HAIs due to all resistant microbes cannot directly be achieved through any national surveillance methods generally used. This data cannot easily be collected at a hospital level either: e.g. prevalence surveys are vulnerable for chance, collection of full cover resistance data is laborious in prevalence survey, and often only half of HAIs are microbiologically confirmed. Therefore, an estimate of the burden is practically the best we can achieve to date. In the future, e.g. enhanced on line HAI reporting e.g. by using compulsory computerized reporting of antibiotic prescribing indications (HAI, community-acquired infection or prophylaxis) and automated combination of microbiological data of causative pathogens might improve the data coverage. To get the most applicable data now, we chose national data sources e.g. to cover estimates of HAIs and deaths. The national prevalence data were quite representative of Finnish acute care hospitals: over 8,000 adult inpatients in all five tertiary care hospitals, and all 15 secondary care hospitals as well as 10 (25% of all) other acute care hospitals took part in the voluntary survey. The resistance data from laboratories also well covered the whole country.

There are, however, several limitations in our estimates. Firstly, we included only seven microbes and four infection types. In the national prevalence survey data, the selected microbes, *S. aureus, Enterococcus* spp.*, E. coli, K. pneumoniae, Enterobacter* spp*., P. aeruginosa and Acinetobacter* spp., caused 48% of all microbiologically conformed HAIs and comprised 34% of all causative microbes [4]. Selected infection types covered 63% of all HAIs. We excluded coagulase-negative staphylococci, as in the ECDC/EMEA report [1], due to its various resistance patterns and relatively low virulence, and pneumococci because a majority of these infections are community-acquired [6]. Rice *et al*. grouped *E. faecalis* and *faecium*, *S. aureus*, *K. pneumoniae*, *A.baumannii*, *P. auruginosa* and the Enterobacter species as the most common nosocomial pathogens that escape the effects of many antibiotics, and used the acronym ESKAPE [7].

Secondly, we used a prevalence survey, not incidence data, to estimate the HAI numbers and distribution. In the prevalence survey, because HAI prolongs the length of stay, severe HAIs, such as BSIs and pneumonias, are likely to be overrepresented in relation to mild infections, like UTIs. In addition, the prevalence data was from 2005, and lengths of stay may have shortened after that, also affecting the proportions of different HAI.

Thirdly, we applied the same ratio of other infections to BSIs for all microbes. In fact, *Enterobacteriaceae* are usually more prone to cause UTIs, and MRSA causes SSI. In addition, the NIDR surveillance data included both primary and secondary BSIs, but the prevalence survey included only primary BSI, which can affect the estimated distribution of infection types, leading to an overestimation of the number other infections by approximately 20%.

Fourthly, infections caused by ESBL producing gram-negatives, especially *E. coli* UTIs, can also be community-acquired, although severe and complicated infections, such as bacteremias [8], may more likely be healthcare-associated. In reports from different hospital settings from the years 1999–2007, the proportion of community-associated BSIs (i.e. in patients with no previous hospitalizations or healthcare-associated risk factors) among all BSIs due to ESBL producing *E. coli* has been low but variable: 1.6% in Italy, 3% in Finland, 19% in Spain but reached 42% in Canada [9-12]. This tendency of having the most bacteremic complications during hospital care also applies to MRSA, although community-acquired skin and soft tissue infections are common [13]. Infections due to other multidrug-resistant microbes in our study can mainly be considered healthcare-associated. We estimate that including all BSIs due to multidrug-resistant bacteria as HAIs leads to an overestimation of HAIs due to these bacteria by another 10%. Finally, the average attributable mortality of 3.2% from the Finnish prevalence survey reflects HAI mortality mostly due to sensitive bacteria, which may lead to underestimation of mortality due to multidrug-resistant pathogens even as much as 50%.

Estimates of the burden of HAI due to antibiotic-resistant microbes are important for those who allocate resources or prioritize the work of an infection control team. In countries with low prevalence of antibacterial resistance, a lot of resources of infection control are often allocated to prevent the spread of resistant microbes, i.e. contact tracing, screening patients for colonisation, applying isolation precautions and occasionally also on decolonization. The emergence of carbapenemase-producing *Enterobacteriaceae* is an example of the importance of timely identifying risk groups for screening and action. However, all infection control resources should not be focused on one microbe or resistance pattern only, such as MRSA or ESBL.

Especially, if the prevalence of antibacterial resistance is high, these narrow spectrum interventions are insufficient to prevent clinical complications. Infection control interventions including evidence-based guidelines, increasingly implemented as “care bundles” and “check lists”, meticulous hand hygiene in all patient care, prudent use of antimicrobials are needed to reduce HAIs in general, along with those caused by multidrug-resistant bacteria. This approach has a greater impact on mortality, morbidity and costs than does screening alone [14,15].

As most HAIs are due to endogenous bacteria, and because e.g. in Finland, up to 94% of HAIs still stem from non-multidrug-resistant bacteria, we should not forget infection-type specific prevention when allocating resources in low prevalance countries either. This is especially important considering, for example ESBL producing bacteria, which are increasingly prevalent in the community and cause preventable catheter-associated bacteremic UTIs in hospital settings.

## Abbreviations

(HAI): Healthcare-associated infection; (ECDC): European Centre for Disease Prevention and Control; (EMEA): European Medicines Agency; (NIDR): National Infectious Disease Registry; (MRSA): Methicillin-resistant *S. aureus*; (VRE): Vancomycin-resistant enterococci; (ESBL): Extended spectrum β-lactamase; (EARS-Net): European Antimicrobial Resistance Surveillance System; (SIRO): The Finnish Hospital Infection Program; (SSI): Bloodstream infection (BSI) and surgical site infection; (UTI): Urinary tract infection.

## Competing interests

The author declares that they have no competing interests.

## Authors’ contributions

MK and OL have designed, analysed and interpreted the data, JO contributed to statistics and AH interpreted FiRe data. All authors read and approved the final manuscript.
